# Applying the COM-B Model to Understand the Drivers of Mistreatment During Childbirth: A Qualitative Enquiry Among Maternity Care Staff

**DOI:** 10.9745/GHSP-D-22-00267

**Published:** 2023-02-28

**Authors:** Muhammad Asim, Waqas Hameed, Bushra Khan, Sarah Saleem, Bilal Iqbal Avan

**Affiliations:** a Department of Community Health Sciences, Aga Khan University, Karachi, Pakistan.; b Department of Psychology, University of Karachi, Karachi, Pakistan.; c Department of Population Health, London School of Hygiene and Tropical Medicine, London, United Kingdom.

## Abstract

**Introduction:**

Respectful maternity care (RMC) during childbirth is an integral component of quality of care. However, women’s experiences of mistreatment are prevalent in many low- and middle-income countries. This is a complex phenomenon that has not been well explored from a behavioral science perspective. We aimed to understand the behavioral drivers of mistreatment during childbirth among maternity care staff at public health facilities in the Sindh province of Pakistan.

**Methods:**

Applying the COM-B (capability–opportunity–motivation that leads to behavior change) model, we conducted semistructured in-depth interviews among clinical and nonclinical staff in public health facilities in Thatta and Sujawal, Sindh, Pakistan. Data were analyzed using thematic deductive analysis, and findings were synthesized using the COM-B model.

**Results:**

We identified several behavioral drivers of mistreatment during childbirth: (1) institutional guidelines on RMC and training opportunities were absent, resulting in a lack of providers’ knowledge and skills; (2) facilities lacked the infrastructure to maintain patient privacy and confidentiality and did not permit males as birth companions; (3) lack of provider performance monitoring system and patient feedback mechanism contributed to providers not feeling appreciated or recognized. Staff bias against patients from lower castes contributed to patient abuse and mistreatment. The perspectives of clinical and nonclinical staff overlapped regarding potential drivers of mistreatment during childbirth.

**Conclusions:**

Addressing mistreatment during childbirth requires improving the knowledge and capacity of maternity staff on RMC and psychosocial support to enhance their understanding of RMC. At the health facility level, governance and accountability mechanisms in routine supervision and monitoring of staff need to be improved. Patients’ feedback should be incorporated for continuous improvement in providing maternity care services that meet patients’ preferences and needs.

## Abstract (precis)

The promotion of respectful maternity care requires addressing the drivers of mistreatment and strengthening the capacity of maternity care staff to provide respectful and rights-based maternity care.

## Teaser

**Key Findings:**

- One of the major drivers of mistreatment during childbirth includes the lack of staff training on interpersonal skills and psychosocial support, which resulted in providers lacking the understanding that patients’ rights also include equity, respect, and satisfaction—and not only the right to high-quality clinical care.
- Providers restricted the presence of birth companions, particularly males, based on providers’ own cultural preferences and comfort, thus denying women a source of support during childbirth.
- Provider’s physical and verbal abuse, neglect, and abandonment of patients, particularly of those from lower castes and minority ethnic groups, in response to patients’ lack of cooperation and compliance was common and justified. Stronger performance monitoring systems and patient feedback mechanisms would help hold staff accountable for mistreatment and contribute to improving respectful maternity care.

**Key Implications:**

- These findings call for a comprehensive intervention that enhances staff members’ knowledge of and positive attitude toward respectful and rights-based maternity care.
- At the health facility level, interventions are needed to strengthen governance, performance monitoring and supervision, accountability mechanisms, and integration of feedback to continually improve maternity care and services.

## INTRODUCTION

Respectful maternity care (RMC) is rooted in a woman-centric approach based on the ethics of and respect for universal human rights. RMC prioritizes the needs of women and newborns during childbirth in every health system.^1^^,^^2^ The 7 universal rights of childbearing women are freedom from harm, consented care, privacy and confidentiality, dignified care, timely and high-quality care, equitable care, and autonomy.^3^

However, a plethora of literature has highlighted a global prevalence of mistreatment during childbirth, particularly in low- and middle-income countries.^4^^–^^7^ In a study in Pakistan, 97% of women reported experiencing at least 1 incident of disrespectful or abusive behavior during childbirth.^8^ Mistreatment during childbirth is likely to have a detrimental effect on the woman-provider interaction and lead to women’s poor childbirth and postpartum experiences.^9^^,^^10^ Short-term adverse consequences of mistreatment may include pain and suffering, a feeling of dehumanization, and fear of childbirth,^11^^,^^12^ while in the long term, a reinforcement of mistrust toward institutional birth may instill in women a preference for home-based births.^1^^,^^13^^–^^15^

Research has tended to focus on women’s experiences of mistreatment during childbirth. However, a growing body of evidence has focused on providers’ perspectives of mistreatment during childbirth and its underlying causes.^16^^–^^21^ Studies have also examined the health system perspective to identify systemic factors of mistreatment during childbirth.^19^^,^^22^^–^^24^ Such studies^25^ have identified several complex, multifaceted factors of mistreatment during childbirth, which can be classified broadly as: individual (prejudice, ensuring compliance for positive outcomes, and stress); sociocultural (normalization of mistreatment and power dynamics); and structural (workload and lack of accountability). It is worth noting that studies investigating reasons for patient mistreatment in general health care identified similar underlying behavioral drivers.^26^^,^^27^

Behaviors are acquired through interaction with the environment; our responses to environmental stimuli shape our actions.^28^ Studies have shown that a lack of health-system support for health care providers reduces their inclination to provide supportive care to patients.^29^^–^^31^ Hence, bringing the insights of behavioral science to bear on mistreatment during childbirth may reveal the psychological dimensions of providers’ behavior, such as perceived notions of what mistreatment is, an instinct to justify mistreatment, and how environmental (social and health system) stimuli influence actions.^32^ Identifying these and other precursors of behavioral manifestations could provide opportunities to design an innovative intervention to address mistreatment during childbirth. An intervention capable of understanding and addressing this complex public health issue requires a behavioral framework.^33^ Moreover, evidence suggests that theory-driven interventions are more likely to be able to change human behavior.^34^^,^^35^

Bringing the insights of behavioral science to bear on patient mistreatment during childbirth may reveal the psychological dimensions of providers’ behavior.

Since patient mistreatment is a behavioral act, likely driven by individual and environmental factors, it needs to be investigated through a behavioral lens. To the best of our knowledge, few studies have adequately explored this complex phenomenon using a behavioral science lens.^21^^,^^36^^,^^37^ Although RMC interventions implemented thus far have shown promising results, concerns remain about their sustainability within the health system.^38^ Efforts to understand the complexity of mistreatment during maternity care should be situated in a defined theoretical framework that can ultimately inform the development of effective interventions for sustainable impact.

The theories of behavior change can be categorized as process models, determinants frameworks, classical theories, implementation theories, and evaluation frameworks.^39^ Given the nature of our project to develop a comprehensive intervention package to promote RMC within public health facilities, we wanted to understand the behavioral drivers of mistreatment during childbirth among maternity care staff using the COM-B (capability–opportunity–motivation that leads to behavior change) model because it lends itself to understanding and/or explaining what influences implementation outcomes. According to the COM-B model, to perform a particular behavior, one must feel both psychologically and physically able to do it, have the social and physical opportunity to do it, and want or need to carry out that behavior more than other competing behaviors.^40^ The COM-B model has been widely used globally, particularly to understand the behavior of health care staff.^41^^,^^42^ Unlike other frameworks, COM-B is relatively simple, yet it allows researchers to distinctively and comprehensively capture the physical, psychological, and social factors that lead to behavioral outcomes^43^ of health care staff providing supportive care to women during childbirth.^39^^,^^44^^,^^45^

In this study, we aimed to understand the behavioral drivers of mistreatment during childbirth among maternity care staff at public health facilities in the Sindh province of Pakistan.

## METHODS

### Study Design and Setting

We used a qualitative descriptive exploratory research design^22^ to carry out this formative study using semistructured in-depth interviews. This study is part of a larger project that aims to develop and test the feasibility of a service-delivery intervention model to promote a culture of support and respect during childbirth in public health facilities.^46^ We conducted the study in all 6 secondary-level public health facilities that provided basic emergency obstetric and newborn care services in 2 districts adjacent to Aga Khan University Karachi: Thatta and Sujawal from Sindh province, Pakistan. These predominantly rural districts report the highest maternal and neonatal mortality due to poor socioeconomic conditions and health system factors. Women are engaged in the informal economy such as working in agriculture fields and in informal domestic labor. Moreover, these districts are categorized in the low human development index strata, where only 17% of women are literate.^47^ The population in both districts combined is about 1.7 million, and 90% of people live in rural areas. The inhabitants are largely Muslim (97%), and 60% of births take place in health facilities.^48^

### Study Participants

A typical maternity team at a secondary-level hospital comprises an obstetrician/gynecologist, who is the in-charge of the department; 1 or more other doctors for each shift; and other clinical staff, including nurses, midwives, or lady health visitors (LHVs). Nonclinical staff include aayas (traditional birth attendants), sweepers, and ward boys (in district headquarters hospitals).

To get a broader perspective, we included 2 categories of participants to interview (N=38): clinical staff (n=23) (including obstetrician/gynecologists, midwives, LHVs, nurses, and technicians) and nonclinical staff (n=15) (aayas, security guards, cleaners, and janitorial staff). We included nonclinical staff because of their involvement in childbirth processes to support clinical staff.^22^ Health facility staff who were working in the maternity wards during the data collection were eligible to participate in the study. The research team conducted individual in-depth interviews to minimize the issue of courtesy bias and concerns regarding a breach of confidentiality by disclosing poor maternity practices.

After receiving permission from provincial and district health authorities and the in-charge of health facilities, the research team approached clinical and nonclinical staff from the obstetrics and gynecology section. The participants were selected purposively according to a predetermined quota from each health facility and cadre according to the designation of providers (Table 1).

**TABLE 1.** Participants Interviewed on Respectful Maternity Care in Sindh, Pakistan

**Maternity Team**	**Participants, No.**
Clinical	
Obstetrician/gynecologist	6
Nurse/midwife/lady health visitor	16
Operation theater technician	1
Nonclinical	
Aaya	7
Cleaner and janitorial staff	7
Security guard	1
Total	38

### Interview Guide Development

The semistructured interview guides were developed based on the COM-B model,^41^ the World Health Organization (WHO) framework of health system building blocks,^49^ and relevant literature.^50^ Owing to the distinct responsibilities and comprehension levels between clinical and nonclinical staff, we developed 2 separate semistructured interview guides (Supplement 1). The interview guides included questions on the health facility structure, quality assurance mechanisms, staff understanding of supportive and respectful care, the patient-provider relationship, and perceptions regarding disrespectful care. Moreover, the interview guides had some open-ended questions and scenarios related to disrespectful care during childbirth at health facilities. These open-ended questions and scenarios were developed based on relevant literature review.^20^^,^^21^^,^^36^^,^^51^^–^^53^ Moreover, we used 2 tools of participatory reflective practices—a timeline and a flow diagram^54^—to collect data on the staff’s daily routine as well as job challenges and mechanisms to address challenges for providing RMC (Supplement 2). The semistructured interview guides were pilot tested on clinical and nonclinical staff at different public health facilities.

### Data Collection

From February 2020 to June 2020, data were collected, but collection was temporarily suspended from March to May 2020 due to the COVID pandemic and lockdown. A total of 38 semistructured in-depth interviews were conducted. All the interviews were conducted in person by 2 trained female sociologists who had prior qualitative research experience related to maternal and child health. The interviewers were trained on research objectives and qualitative data collection before field activities. Thirty-four interviews were conducted in health facilities and 4 interviews were conducted at participants’ homes at their request. Interviews were conducted in a separate room where only the research team and interviewee were present. The interviews were conducted in the local Sindhi language and were audio-recorded after participants gave their written consent. After each interview, the research team debriefed to reflect on the participant’s responses. On average, each interview lasted approximately 45–60 minutes. The data collection concluded when the research team collected no new information. The research team discussed the data saturation point during the analysis of interview notes of 38 interviews. When the researchers did not find new information and themes from the data, the researchers decided to conclude the further data collection.

### Data Analysis

All the recorded interviews were transcribed directly into English language by linguists who had command of Sindhi and English languages and had experience translating health-related interview transcriptions. To ensure data quality, 2 authors reviewed (WH, MA) all transcripts using both written notes and audio recordings. A deductive content analysis approach^55^ was used based on the COM-B model. After several deliberations within the research team, the model was contextually adapted in accordance with the issue of RMC. We first outlined the operational definitions of the broader framework components (capability, motivation, and opportunity) and their subcategorization (Figure). Thereafter, we reviewed the transcriptions and made minor adjustments to the definitions where necessary. The data analysis process included an intensive review of all transcripts by 2 authors (MA, WH). Moreover, field notes were also reviewed for in-depth understanding and contextualization of the participant’s quotes. The data were sorted and analyzed through NVivo version 11.0 software. Two authors (MA, WH) generated an exhaustive list of relevant codes of corresponding themes and subthemes by using a COM-B model. These themes were used to develop a theoretical matrix that all authors reviewed and from which subthemes emerged. The authors (MA, WH, BK, BIA) discussed the interpretation of themes and subthemes before reaching a consensus. Similar codes or recurring participant statements were omitted during article preparation.

**FIGURE:**
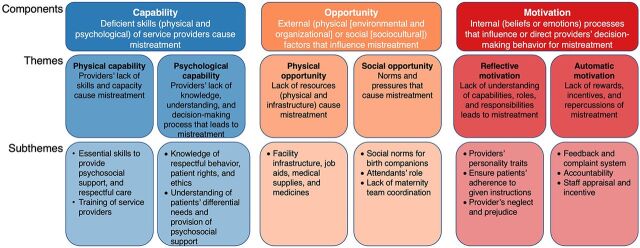
Adaptation of the COM-B Model in the Context of Mistreatment During Childbirth and Emerging Subthemes Abbreviation: COM-B, capability-motivation-opportunity that leads to behavior change.

### Ethical Approval

The Aga Khan University Ethics Review Committee (Reference ID: 2019-1683-5607) and the London School of Hygiene and Tropical Medicine Institutional Review Board (Reference ID: 17928) provided ethical clearance for this study.

## RESULTS

Nearly all the participants (95%) were female. Participants’ mean age was 39 (standard deviation ±7.3) years. Almost two-thirds of the participants (61%) were clinical staff. Most of the staff worked morning or evening shifts (Table 2).

**TABLE 2.** Characteristics of Study Participants

	No. (%)
Sex	
Female	36 (95)
Male	2 (5)
Age, years, mean (±SD)	39 (±7.3)
Health worker cadre	
Clinical staff	23 (61)
Nonclinical staff	15 (39)
Total experience, years, mean (±SD)	13.4 (±8.3)
Working shifts	
Morning	16 (42)
Evening	12 (32)
Night	4 (11)
Rotation	6 (11)

Abbreviation: SD, standard deviation.

We categorize our findings into the 3 COM-B components: capability, opportunity, and motivation.

### Capability

Capability refers to both the physical capability, which includes providers’ skills and capacity to provide RMC, and the psychological capability, which includes providers’ knowledge, understanding, and decision-making processes to provide RMC.

#### Physical Capability

We defined psychosocial support as staff providing psychological and social resources to the pregnant woman to help her overcome the physical, mental, and emotional challenges she may face during labor and childbirth. Not all the clinical staff knew about the importance of providing psychosocial support during childbirth. When asked about providing psychosocial support to patients, some clinical staff equated this to speaking politely with patients.

*We speak softly with patients and try to provide optimal care during the stay of patient at the facility*. —Nurse, Thatta

When asked about providing psychosocial support to patients, some clinical staff equated this to speaking politely with patients.

The clinical staff reported that nonclinical staff usually misbehave with patients and that they need orientation on interpersonal communication skills.

*Lower staff doesn’t even have a common sense to talk with the patients. They misbehave and scold the patients all the time … They even degrade the patients by pointing out that you smell so bad and sit outside because you are bleeding. They should not treat patients in this way.* —LHV, Thatta

However, when we interviewed the nonclinical staff, they leveled accusations that clinical staff misbehaved with patients. It is evident that the entire staff misbehaved with patients, and no one took responsibility for their disrespectful behavior.

We also found that almost all participants reported that they did not receive specific trainings on interpersonal behavior and RMC. A few clinical staff reported that they attended trainings that focused on clinical care, patient safety, and family planning. Because more focus is given to providing standard clinical practices for improved health outcomes, trainings related to providing supportive care and psychosocial support are largely not offered at childbirth health facilities.

*Refresher courses related to clinical care are conducted occasionally at our health facilities. However, training on behavioral change is never provided since I joined public service. There is no mechanism on providing supportive care during childbirth in our health facilities. However, all service providers have their own ways of doing things.* —Nurse, Sajwal

Some nonclinical staff mentioned that they never received specific training to improve their interpersonal and supportive care skills.

*Ever since I am in service, I never had any training. I have requested many times to my seniors so that if we are lacking in anything, it can be improved by training. Our seniors, doctors, and nurses have trained us from starting to prepare trolley and how to clean instruments. We never had training on supportive care and how to behave with maternity patients.* —Aaya, Thatta

In contrast, a clinical staff member with more than 10 years’ experience reported that they do not need any training related to supportive care because they are qualified physicians and do not need any further training on supportive care of patients.

*Guidelines, manuals, and training are for unqualified [quacks] health care providers. I am a qualified physician, and I am very clear in my work… I know what to do during the job. Consultants don’t need the trainings for such purposes.* —In-charge, Thatta

#### Psychological Capability

The lack of understanding of patients’ rights, respect, and support was the major factor in mistreatment during childbirth. Clinical staff reported that they consider patients’ rights to be providing high-quality care, including having available doctors at health facilities, performing proper examinations, and having adequate stock of drugs and other clinical supplies. However, providers’ respectful behavior toward patients, equity, privacy, politeness, and patient satisfaction were not considered core components of patients’ rights.

*The medicine which we receive should reach to the patients… that are the patients’ rights*. —Midwife, Thatta

Clinical staff considered patients’ rights to be having available doctors at health facilities, performing proper examinations, and having adequate stock of drugs and other clinical supplies.

However, a consultant shared a similar viewpoint that the availability of doctors for timely care at health facilities is the primary right of patients.

*The most important thing is the availability of doctors at health facilities. Patients come to hospitals to seek treatment from physicians and usually physicians do not attend and see the patients in many public hospitals. That’s the main thing for patient right if physician attend the patients at health facilities.* —In-charge, Thatta

We found that clinical staff define respect as speaking courteously and showing respectful behavior to patients. However, most clinical staff members expected respect to be reciprocated between staff and patients.

*Respect is necessary and a bilateral phenomenon between provider and patient. As care providers, we should respect patients and they [patients] should respect us to get back more respect.* —Nurse, Thatta

Clinical staff considered patients’ rights to include taking care of them and fulfilling their material needs with integrity. Moreover, they viewed patient support as providing clinical information to motivate and guide patients.

In meeting patients’ differential needs, most clinical staff reported that they understood patients’ needs and provided support based on their past experiences. Furthermore, they explained psychosocial support in terms of providing medicine or giving money to those who cannot afford care.

Participants reported that physically and mentally disabled women usually delivered their babies at home. However, a few providers shared that they were equipped to fully support disabled women during pregnancy.

*Most of disabled women deliver babies at home. However, some dumb and deaf patients come for delivery in our hospital. In such situation, we invite their attendants in the labor room. Because we can’t understand their ambiguous language and gestures.* —In-charge, Thatta

*Some patients have problem with their hands or legs, and they need help during delivery and childbirth. We ask their attendant to come and hold their legs and give them support.* —Nurse, Thatta

### Opportunity

Opportunity refers to external factors that influence mistreatment during childbirth. These could include physical opportunities, such as lack of resources and infrastructure, and social opportunities, such as unclear social norms, pressures, and lack of peer relationships that cause mistreatment during childbirth.

#### Physical Opportunity

The absence of mechanisms to maintain privacy and confidentiality because of a lack of physical infrastructure and essential equipment was a major concern at health facilities.

*We have 1 labor room that has 10 beds without separators. Moreover, we have 1 operation room with 3 beds and all surgeries are being conducted without separators.* —Aaya, Thatta

Moreover, a shortage of beds in the labor room was also common. An LHV raised her concern about the lack of physical infrastructure to provide quality care.

*We have 1 labor room with 3 beds. We manage almost 100 deliveries every month and sometimes it is difficult to allocate beds to the patients.* —LHV, Thatta

Both clinical and nonclinical staff highlighted that guidelines related to RMC were not available at health facilities. Some facilities had educational posters mounted on the walls that explained standard procedures for clinical care; however, guidelines related to RMC were missing.

*We have posters about eclampsia, postpartum hemorrhage, and COVID-19 in wards and operation theaters. The main thing is to protect ourselves from infection. During surgery, I repeat the procedure to mentor juniors and tell them standard procedure to control the infection.* —Nurse, Thatta

Staff mentioned that guidelines related to RMC were not available at health facilities.

Moreover, clinical staff mentioned that the limited supply of drugs in the health facility is a major barrier to providing care to all patients.

*We have limited supply of drugs and patients who came to our facility demand medical supply from us. Most of drugs get out of stock immediately and we cannot give drugs to patients to satisfy them.* —LHV, Thatta

#### Social Opportunity

A birth companion is a trustworthy person (e.g., her husband, family member, or friend) who provides support to women during labor and childbirth. Participants reported that they perceived the birth companion’s role was to support women in tasks, such as preparing admission/discharge documents, accompanying them for medical tests (e.g., ultrasound), and getting prescription medicines if they were not available in the hospital. Both clinical and nonclinical staff highlighted that managing birth companions was a major challenge because they cause a lot of problems and delays in providing care to patients. However, clinical staff have a duty to be respectful of the supportive and companion care. Clinical staff pointed out that companions sometimes become aggressive and commit violence at the facility.

*[Companions] do physical violence with our staff. Once a female patient was expired in the morning, her family members broke the glasses of windows of labor ward. They hit the duty doctor. They also verbally abuse us if they do not get medical supplies. [Companions] also make videos of empty counter and saying that nobody is available at labor ward to harass us.* —Nurse, Thatta

Clinical staff reported that patients sometimes arrive with several companions, which poses another challenge to accommodate and manage these companions at the facilities.

*A maternity patient comes at health facility with whole family including elders and children. This is a custom in our society. However, when we need a donor, then nobody donate blood.* —LHV, Thatta

When asked about engaging birth companions, participants reported that only female companions were allowed to accompany women during labor and, depending on the situation, some women were allowed to have companions in the delivery room. Male companions were not allowed in the maternity ward nor in the labor room because of the privacy needs of other patients who were in the same labor room. In fact, clinical staff reported they felt uncomfortable when male companions were present while women were undressed at the time of delivery.

*In my opinion every woman has a certain level of privacy that can’t be comprised. Even their husbands should not see them in that state [naked]. Moreover, I [CMW] and my support staff are also present there [delivery room] … They [male companions] are their husbands not ours… Woman is nude in front of their husband and we [female staff] are also standing there. I will not allow such unethical practices at my health facility.* —Midwife, Thatta

The presence of a male companion at the time of delivery was not a norm at health facilities.

*Delivery can be done without the presence of husbands. If there is any separate setup, then her husband cab shows up there. In my opinion, it is important for husband to leave the place during delivery in order to perform duty by the care providers. However, husband or male member can present during the labor.* —In-charge, Thatta

The other major social issue was staff working relationships and team coordination. Participants noted that although the working relationship between colleagues and supervisors was good, there was a lack of coordination and mutual respect between nonclinical and clinical staff. Nonclinical staff expressed concerns about clinical staff’s rude behavior, particularly of senior doctors, while they cannot manage the companions at health facilities.

*My duty is to manage people in [the outpatient department] so that doctors and nurses can see patients and perform their work easily. If maternity patients and attendants do not listen to us, then physician insult and abuse us…. This is not right to insult us in front of patients.* —Aaya, Sujwal

### Motivation

Motivation refers to the processes that influence or direct clinical staff’s decision-making behavior for RMC. Reflective motivation is the lack of provider understanding about capabilities, roles, and responsibilities that lead to mistreatment. Automatic motivation is the lack of rewards, incentives for respectful care, and lack of repercussions for mistreatment.

#### Reflective

Clinical staff reported that their colleague’s repeated rude behavior toward patients was accepted as their personality attribute.

*There is a doctor in our shift that gets so angry on patients on their small actions. Her behavior remains rough while treating the patients at labor room. When she gets angry with patients, then we try to keep our attitude good, so the patient doesn’t get too nervous.* —LHV, Sujwal

Participants reported that psychological and physical abuse was common during childbirth, particularly at secondary-level health facilities. Violence often occurred when patients did not follow instructions, patients did not cooperate, many people were at the facility, and supplies were not available. Moreover, clinical staff reported that patients only followed instructions when they dealt sternly with patients.

*I have observed that if a woman has labor pain and the baby is about to out, but the push of woman is not efficient… and baby’s life may be critical. In such circumstances, staff members can shout and slap to save the life of baby.* —In-charge, Thatta

*Women who cry and do not cooperate… then we have no choice than to scold and beat them. We hold them [women] in the position so that they can deliver baby easily. Baby’s life must be saved in any case. Babies die in the womb of women who do not follow the instructions of providers.* —Midwife, Thatta

Participants reported that providers neglected and abandoned patients who did not follow their instructions during delivery.

*At the time of delivery, we counsel women and tell them how labor pain gets increase. We leave them for some time if they don’t follow our instructions. After some time, women understand that they have to deliver a baby and then we start treatment.* —LHV, Thatta

Participants reported that providers neglected and abandoned patients who did not follow their instructions during delivery.

Moreover, nonclinical staff also reported that it was difficult to provide respectful care to some patients because of some patients’ physical appearance. However, clinical staff did not mention any discrimination based on patients’ physical appearance.

*We pray that [retracted] caste people don’t come here. Doctor feels difficulty to give assistance to them because of bad smell from their body. They don’t come prepared for delivery.* —Housekeeping staff, Sujwal

#### Automatic Opportunity

When the maternity teams discussed providing emotional (e.g., consoling distressed women) and instrumental support (e.g., giving money out-of-pocket), they mentioned that they did this because of the praise and prayers they received from women and their companions. This praise gave them emotional satisfaction, reenergized them, and gave them a sense of accomplishment.

Most participants knew that showing respectful care added to their reputation in the community because women shared their positive experiences with other women in the community and even mentioned maternity team members by name.

There was no patient feedback and complaint system in place at health facilities. Some clinical staff mentioned that they reported issues and complaints to senior doctors or health facility in-charges who took action and resolved the issue. There was no mechanism for patients to submit written feedback or suggestions regarding maternity care at health facilities.

*The documentation of complaints and feedback from patient are unimaginable thing in our facilities… This system based on verbal communication. We resolve issues on the spot. No one knows about such existence of formal accountability system for complaints.* —LHV, Sujwal

*There is no proper monitoring system here. I am the only one who listens to all patients and staff. People come to me and bring up complains about the ward staff. For example, staff didn’t empty urine bag. Sweeper demands 50 rupees from the patients to empty the urine bag…. All these things disturb the patients. When they bring the complaints then I talk to the sweepers. If she didn’t understand, then I scold her.* —Nurse, Thatta

Nonetheless, clinical staff felt that a patient complaint mechanism could help keep staff accountable for their behavior toward patients. Knowing that patients had an opportunity to make a formal complaint would ensure that staff would avoid any disrespectful or abusive behavior toward patients.

*If we introduce a patient complaint system in health facility, it will make maternity staff more cautious about how they behavior with patients. They will have a fear that if they do something wrong with patients, they [patients] could go and lodge a complaint against them.* —LHV, Thatta

Informal cash payment was common among nonclinical staff (aaya and janitorial staff) because of the lack of accountability at the health facility and staff’s lower salaries. Nonclinical staff asked for money at the delivery room from birth companions immediately after childbirth.

*It is common to give cash to aaya and sweeper and they [attendants] give cash on their own will. Our staff does not ask for cash themselves. However, we sometime say that these staff workers are poor so give them some cash.* —Midwife, Sajwal

Although health facility administrators visited health facilities to regularly monitor staff activities, they usually focused on the availability of medical supplies and medicine, staff attendance, and cleanliness of the health facility instead of staff behavior.

*Mostly they [external monitors] come in the morning shift, such as district deputy commissioner and assistant commissioner. They put more focus on cleanliness, duty rosters, dress code of staff, and availability of medicines.* —Aaya, Thatta

According to participants, a systematic mechanism to review staff performance did not exist at health facilities. Although a formal annual staff appraisal system existed, the process did not consider the clinical staff performance with respect to RMC. However, occasionally, senior staff conducted meetings to resolve issues. Most maternity team members were motivated to perform their job responsibilities, but they expressed the need for formal appreciation and recognition from administrative leadership for those who exceeded expectations at work.

*Here, nobody will appreciate you; no matter how hard you work. They just want to work, work and work, and don’t ask anything… appreciation will certainly higher your morale so that you work harder.* —Nurse, Thatta

Most maternity team members were motivated to perform their job responsibilities, but they expressed the need for formal appreciation and recognition from administrative leadership.

## DISCUSSION

Our formative study used the COM-B framework to examine the behavioral drivers of mistreatment during childbirth at public health facilities in Pakistan. Supplement 2 presents a summary of key findings on the drivers of mistreatment during childbirth by both clinical and nonclinical staff. Our research adds to the findings of recent systematic reviews about health professionals’ understanding of mistreatment during childbirth. We systematically examined behavioral drivers of mistreatment during childbirth using an established theoretical framework and deliberated on their interconnectedness. More importantly, our study also provides additional insights concerning systemic issues that trigger mistreatment during childbirth.^25^

### Capabilities of Maternity Care Staff and Mistreatment

Staff, particularly nonclinical staff, lacked knowledge about respect, rights, privacy, and equity with respect to patients. Patients’ rights were understood in terms of tangible items, such as the availability of drugs and supplies and the presence of a doctor at the health facility. Respect for patients was perceived as conditional and reciprocal—to be earned by the patient’s respectful attitude toward staff. This perceived notion of respect may be a precursor to the mistreatment of pregnant women. Many staff cited instances of mistreatment as a reaction to patients’ disobedience or abusive behavior.^5^^,^^18^ Both clinical and nonclinical staff confirmed that women were mistreated during childbirth at health facilities, but they blamed each other for the manifestations of such mistreatment.

Respect for patients was perceived as conditional and reciprocal—to be earned by the patient’s respectful attitude toward staff.

Psychological distress is commonplace during childbirth. Hence, WHO recommends providing emotional support during intrapartum care. Our study highlights a lack of realization among clinical staff of the importance of identifying and addressing the differential physical and mental health needs of maternity patients. Clinical staff focused primarily on clinical conditions and the clinical needs of patients to achieve improved birth outcomes, most likely because they never received training on systematically screening patients for differential needs and for psychosocial support during childbirth. Because of the high level of poverty observed among patients in the study districts, most clinical and nonclinical staff identified free medicine and financial help as constituting patients’ needs beyond physical health care. With improved knowledge and skills, clinical staff would be able to identify women’s emotional needs and determine the right psychosocial support to meet those needs, likely resulting in reduced distress and better birthing outcomes.^56^ There is a clear need for clinical and nonclinical staff to be trained on the integral domains of WHO’s framework for maternal and newborn care: dignity and respect, effective communication, and emotional support.^57^ Training maternity staff on behavioral change has shown improvements in their understanding of RMC.^50^

### Opportunities in the Contexts of the Health Facility and System

We found that a range of systemic and broader social factors formed the foundations of mistreatment during childbirth. The presence of male companions was highly restricted at the time of delivery because of the cultural and religious values of both providers and patients in maintaining the privacy of (other) patients. Interestingly, female clinical staff voiced discomfort about their own privacy being compromised in the presence of male companions at the time of childbirth. The husband’s role is widely recognized as a source of general and emotional support to women during pregnancy and childbirth. However, different studies have pointed out that male involvement in childbirth is restricted because of facility-related constraints, an unwelcoming health system, and cultural inclinations.^58^^–^^61^ The engagement of companions during maternity care was further limited by negative perceptions of companions as an unnecessary interruption in care provision. Janitorial staff felt that companions were a burden on them as they created a mess on the wards that they needed to clean up. In such situations, they felt the need to assert their authority to ensure compliance with health facility rules, often resulting in mistreatment.^62^ Similar observations were made in studies conducted in African countries.^7^^,^^18^^,^^32^ However, female birth companions generally enjoyed cultural acceptance by both patients and staff and were widely permitted in health facilities.

Both clinical and nonclinical staff highlighted the unavailability of basic infrastructure, such as privacy screens/curtains in labor rooms. Various studies have pointed out that in the most resource-limited settings, many patients share labor rooms.^63^^–^^65^ In such environments, women experience exposure to other patients, male visitors, and staff who are not attending to them as undignified, inhumane, or shameful.^7^ Studies from South Asian and African countries also report that women experience compromised auditory and visual privacy, which resonates with our findings.^7^^,^^65^ Also, although health facilities had guidelines related to clinical procedures, they lacked guidelines or job aids related to RMC. This may explain clinical staff’s predominant concern with the technical aspects of clinical care to ensure good outcomes while neglecting the elements of RMC. Administration focused on material management, staff attendance, and facility cleanliness while giving relatively little attention to patient-provider behavioral interactions.

Clinical staff had clearly defined working relationships and coordination mechanisms in place. However, there was a gap and a degree of conflict in working relationships between clinical and nonclinical staff. This reflects the power dynamics of the existing hierarchy. A sense of inferiority, feeling of powerlessness, and lack of coordination among nonclinical staff might cause frustration and a tendency to assert what power they feel they have on the patient, which may result in mistreatment.^52^

### Motivation and Mistreatment

Physical and verbal abuse were commonplace during childbirth, more so in cases of difficult deliveries and patients’ noncompliance with staff instructions. Clinical staff justified their abusive behavior and use of threats by claiming they acted in the interests of the mother and newborn to save their lives. In addition, clinical staff used neglect and abandonment as leverage to ensure patients’ compliance. Other qualitative^5^^,^^11^^,^^18^ and quantitative^4^ studies have found that mistreatment and abuse are commonplace during childbirth in low- and middle-income countries. The acceptance of and lack of willingness to discuss such actions are indicative of both the normalization of this behavior^11^^,^^66^ and a lack of any process to monitor it.^53^

Clinical staff justified their abusive behavior and use of threats by claiming they acted in the interests of the mother and newborn to save their lives.

Staff, particularly nonclinical staff, harbored ethnic, tribal, and caste-based prejudices resulting in patient abuse. Such behavior decreases patients’ trust in both health care providers and the health system.^67^ Studies from India and Pakistan reported that women who belonged to lower-caste and minority-ethnic groups were more likely to be mistreated.^20^ Providers’ negative attitudes and abusive behavior undermined the well-being of affected maternity patients and were a disincentive for women to seek health care.^9^ Patients commonly made informal cash payments to nonclinical staff to ensure the provision of certain facilities and services to patients, birth companions, and visitors. Cash payments, particularly after childbirth, are widely accepted and viewed as a gift or a stipend.^68^^–^^70^ Having rigorous monitoring, accountability, and patient feedback mechanisms are important strategies to improve RMC.^71^ The health facilities in this study had no formal mechanisms for patients to provide feedback about their care experiences, but patient feedback plays a pivotal role in improving the quality of health care.^72^

### Strengths and Limitations

A major strength of our study was the use of a behavioral framework to holistically understand the drivers of mistreatment during childbirth in a health system context and to inform the development of interventions to address this issue. The inclusion of nonclinical staff was another strength of the research that enabled us to understand mistreatment during childbirth across various cadres of the maternity team.

Our study had a few limitations. First, we did not consider patients’ perspectives and experiences. Patients’ and community members’ voices need to be heard to fully recognize and address mistreatment during childbirth. A triangulation of our findings with women’s perspectives would have helped validate some claims that staff made about pregnant women and their companions. We have previously conducted studies on women’s experiences of childbirth in health facilities and reported our findings.^20^^,^^36^^,^^73^ Second, the sensitive nature of the topic may have introduced a bias of social desirability whereby providers might have felt reluctant to speak, resulting in the under-reporting of disrespectful and abusive behaviors prevalent at the health facility. Third, study participants were selected from secondary-level health facilities in 2 districts of Sindh. This may limit reflections on our findings to just secondary-level facilities because service delivery processes in primary- or tertiary-care settings differ.

## CONCLUSIONS

We identified factors across 3 components of behavioral drivers. Limited training opportunities resulted in providers’ lack of knowledge regarding patients’ rights and limited skills to recognize their differential needs, which were major determinants of mistreatment during childbirth. Our findings point to the need for capacity building of maternity staff on respectful and rights-based maternity care to address mistreatment during childbirth. By clarifying essential values, these behavioral trainings should also focus on changing the mindset of providers on matters of prejudice and on the assumption that respect is a reciprocal phenomenon.

Inadequate infrastructure, unavailability of RMC guidelines compounded by social norms for birth, and lack of coordination among the maternity team were causes of mistreatment during childbirth. A further aggravating factor was the prohibition of a male birth companion because of health facility culture and local norms. To improve coordination between clinical and nonclinical staff, the organizational culture needs to be sufficiently generous and sensitive to mitigate the power dynamics of the maternity staff hierarchy.

Finally, the providers’ personality traits, mindsets that patients are difficult and uncooperative, normalization of mistreatment, and lack of accountability and governance mechanisms were identified as motivational drivers of mistreatment during childbirth. Therefore, at the health facility level, governance and accountability mechanisms need to be improved and include routine supervision and monitoring of staff and integration of patients’ feedback for ongoing improvement in making care more responsive to patients’ needs.

## Supplementary Material

GHSP-D-22-00267-Supplements.pdf

## Notes

**Peer Reviewed**

**First published online:** January 23, 2023.
