# Supplementary material for: Applying the COM-B Model to Understand the Drivers of Mistreatment During Childbirth: A Qualitative Enquiry Among Maternity Care Staff
Source: Glob Health Sci Pract. 2023 Feb 28;11(1):e2200267. doi: 10.9745/GHSP-D-22-00267 (PMC9972373; doi:10.9745/GHSP-D-22-00267)
Supplement: GHSP-D-22-00267-Supplements.pdf [file GHSP-D-22-00267-Supplements.pdf]

Supplement to: Asim M, Hameed W, Khan B, Saleem S, Avan BI. Applying the COM-B model to understand the drivers of mistreatment during childbirth: a qualitative enquiry among maternity care staff. Glob Health Sci Pract. 2022;10(6):e2200267. https://doi.org/10.9745/GHSP-D-22-00267

Supplement 1. IN-DEPTH INTERVIEW GUIDE FOR MATERNITY STAFF

S.no	Questions	Responses	Skip
**General Information**			
101	Date of interview	______ / _______ / _____________<br>dd mm yyyy	
102	Name of district	Thatta 1<br>Sujawal 2	
103	Name of TALUKA / Tehsil		
104	Name of Union Council		
105	Name of health facility		
106	Name of health facility / Hospital		
107	Type of study participant	Administrative staff 1<br>Clinical staff 2<br>Non-clinical staff 3	
108	Sex	Male 1<br>Female 2	
109	Age (in years)	Years [ ][ ]	
110	What is your designation at this health facility?	Medical superintendent 1<br>In-charge- Obs/Gyne Section 2<br>Medical Officer 3<br>Nurse 4<br>Midwife 5<br>Sweeper 6<br>Aaya 7<br>Security guard 8<br>Technician 9<br>Others specify____________________ 10	
111	How long you have been working in this health facility?	Years [ ][ ]	
112	What is the duration of your total professional experience?	Years [ ][ ]	
113	Highest level of education completed?		
114	In which shift do you work?	Morning 1<br>Afternoon 2<br>Night 3<br>Others specify________________________	
115	Interview start time	|___|___|:|___|___|	
116	Interview end time	|___|___|:|___|___|	

Interview guide with health facility staff

No.	Questions	Probe
**Q1**	First, I would like to ask you some questions about the health facilities – its infrastructure, staffing, kind services that are offered here, patient volume, available guidelines, and how things are routinely performed and managed here.	
	MAIN THEME: STRUCTURE OF HEALTH FACILITY AND ROUTINE OPERATIONS	
101	What maternal and child health services are offered in this Obs/Gyn department?	*Ask separately about maternal and child services*
102	How many rooms and beds are available? What is the usually average volume of birthing women per month?	**Ask separately about:**<br>• Can you tell me the number of normal deliveries conducted in a month?<br>• Can you tell me the number of C-Section deliveries conducted in a month?<br>• If fixed, what days are fixed for C-Sections?<br>• What days of a month you have high and low volume of births?
103	Can you describe team composition or organogram of Obs/Gyn department?<br>**Draw organogram**	**Ask about:**<br>• What is the number of total staff members?<br>• Who are the supervisors at each level?<br>• How the work shifts are organized?<br>• Who has the decision making authority at each level? And what kinds of decision she can take? Please give example.
104	What kind of clinical and non-clinical trainings are provided to the staff here during the last 2 years?	**Probes:**<br>• Can you tell me the objective of each training?<br>• Who conducted those trainings?<br>• How many times the training was conducted and for whom?<br>• What was the duration of each training?
105	Can you describe how patients' –information is collected and stored?	**Probes:**<br>• Please describe in detail about the type of information that is collected at each stage women go through from admitting to the hospital and all the way to discharge.<br>• Can you tell me about the format/forms that are required to be filled at each stage and who filled these forms?<br>• Where these forms/patient files are stored and retrieved?<br>• If any, what data are entered into the computer?
106	Can you tell me how the patients' information is used for: a) decision making to ensure that appropriate / needed care is provided to the woman; and b) to improve the overall performance to this department?	**Probes:**<br>• Can you give an example how patient data were used to provide appropriate / needed care to the patient?<br>○ Who takes the decision?<br>○ How the decision is taken?<br>• Can you give an example how patient data were used to improve the performance to health facility?

		○ Who takes the decision?<br>○ How the decision is taken?
	**Sub-theme: Quality assurance**	
107	**Now I would like to ask some questions the mechanisms that are in place to ensure quality of services that are provided to the patients. Please note that I'm particularly interested in knowing the care that is provided to women who come here for childbirth.**	
108	What kind of guidelines are available for service provision?	Clinical care; non-clinical (communication, respect, support etc.), record keeping
109	How do you ensure that maternity care services are being provided in accordance with standard operating protocols (SOPs)?	Clinical care:<br>• Are you trained on these SOPs?<br>• Who is responsible for ensuring that everyone adheres to the SOP?<br>• How frequently do they monitor?<br>• How does s/he makes sure compliance (what does s/he look at)<br>• What actions are taken in case of non-compliance?<br>• Who is responsible to address gap in implementation of routine service delivery?<br>• Is there any forum where shortcomings are discussed (e.g. performance review meeting?) If yes, please tell us about it?<br>Non-clinical:<br>• Do the SOPs cover how staff should behave with the patient (e.g. respect, friendliness, effective communication etc?)<br>• How it is ensured in routine practice<br>• What actions are taken in case of non-compliance?
110	What is the role of district health office to ensure quality maternal care in this hospital?	• How the support is provided?<br>• Who is responsible?<br>• How effective it is?
**Q2**	**Theme: Understanding of key concepts**	
	I would like to gauge your understanding of few terms/concepts that are used in healthcare. **I will show you a picture and will read out few words, and will ask you to explain what you understand by them. Let me remind you that there is nothing right or wrong. We only like to know your opinion.**	
201	Instruction: Please show this picture to the respondent and ask the following questions:<br>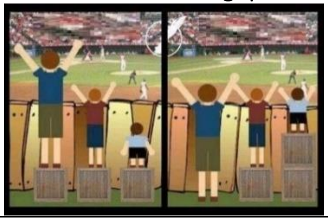	• Can you describe what do you see in this picture?<br>• Can you share some examples from your daily life to explain this picture?<br>• How do you relate this to the care that is provided to women in the labour room and maternity wards?<br>• Do you think women come to this facility for childbirth have varying needs?<br>• How these differential needs are addressed during care provision?<br>• What challenges do you face in addressing these varying needs?

202	How would you define the word 'respect'?	• Can you share some examples from your daily life to explain this word?<br>• How do you relate this to the care that is provided to women in the labour room and maternity wards?<br>• How do you ensure that maternity care is provided in a respectful manner?<br>• What challenges do you face in providing maternity care in respectful manner?
203	What you understanding by the term 'support'?	• Can you share some examples to explain the term in your daily life?<br>• How to you provide support to your family – wife, mother, children?<br>• How do you relate this to the care that is provided to women in this hospital?<br>• What kinds of support is provided to the women during maternity care?
204	What do you understand by the term "medical ethics?"	• Can you share some examples to explain the term<br>• How do you relate this word with the maternity services provided at this health facility? Give some examples<br>• How to you provide support to your family – wife, mother, children?
205	What do you understand by the term "patients' rights"?	• Can you share some examples to explain the term<br>• How do you relate this word with the maternity services provided at this health facility? Give some examples?<br>• How do you ensure that rights of patients are ensured during maternity care?<br>• What challenges do you face in providing right-based maternity care?
206	In your opinion, what are the rights of patients who come to this facility for childbirth?	Share some examples<br>- Right to information (effective communication)<br>- Consented care (informed consent)<br>- Autonomy (respecting women's choices)<br>- Confidentiality / privacy<br>- No verbal abuse (shouting, yelling, threating etc.)<br>- No physical abuse (beating, slapping etc.)<br>- No sexual abuse<br>- No neglect or abandonment (e.g. ignore, delay, refusal)<br>- Non-discriminatory care (e.g. poor care for disabled etc.)<br>- Continuous/supportive care (e.g. companionship)
**Q3**	Theme: Roles and responsibility of staff	
	Now I would like to specifically ask about yourself and your role in this health facility	
301	What is your role / job responsibilities in this hospital?	- Can you describe as per your official job description<br>- What additional work do you do apart from your job description (if any)
302	Describe what your typical day at this health facility looks like. **DAILY ROUTINE – encourage to draw a timeline figure**	**Instructions:** Please ask the respondent to describe each activity in detail from the point they enter and till s/he leaves the health facility.

303	What do you like about your work? Things that keep you motivated?	Why do you like these things?
304	What part of your job do you like the least OR you do not like?	Why do you like it the least?
305	What are the challenges you face in your day-to-day work?<br>First, Can you tell me about the challenges related to your co-workers?	Probes:<br>- Why do you consider these as a challenge?<br>- Why do you think you face those challenges?<br>- How do you deal with or overcome these challenges?<br>- Are there any institutional guidelines/mechanisms to deal with those challenges?<br>- And how effective they are in resolving the issue?<br>- What type of relationship with your colleagues?<br>- In your opinion how can build a better working relationship with your colleagues?<br>-
306	Can you tell me about the challenges related to your supervisors?	- Why do you consider these as a challenge?<br>- Why do you think you face those challenges?<br>- How do you deal with or overcome these challenges?<br>- What are some of the key challenges in implementation of these guidelines?Are there any institutional guidelines/mechanisms to deal with those challenges?<br>- And how effective they are in resolving the issue?<br>- How do you usually official terms with your supervisor or senior?<br>- What do you think how can you improve your working relationship with your supervisor or senior?
307	Can you tell me about the challenges related to your patients?	- Why do you consider these as a challenge?<br>- Why do you think you face those challenges?<br>- How do you deal with or overcome these challenges?<br>- Are there any institutional guidelines/mechanisms to deal with those challenges?<br>- And how effective they are in resolving the issue?
308	Can you tell me about the challenges related to your attendants?	- Why do you consider these as a challenge?<br>- Why do you think you face those challenges?<br>- How do you deal with or overcome these challenges?<br>- Are there any institutional guidelines/mechanisms to deal with those challenges?<br>- And how effective they are in resolving the issue?
309	Can you tell me about the challenges related to the systems like record keeping?	- Why do you consider these as a challenge?<br>- Why do you think you face those challenges?<br>- How do you deal with or overcome these challenges?<br>- Are there any institutional guidelines/mechanisms to deal with those challenges?<br>- And how effective they are in resolving the issue?
4	**Sub-theme: Provider burnout**	

401	Often due to amount or nature of work we become physically and mentally exhausted. As a result of which, we lose interest in our work or felt that the efficiency has reduced. This concept is called "Burnout".<br><br>Did you ever experience this?	
402	When did that happened to you?<br>If your opinion, what the reason for that?	Probe:<br>- Was your supervisor or senior responsible for your burnout? Please explain in detail with example.<br>- Was your co-worker responsible for your burnout? Please explain in detail with example.<br>- Were the patients or their attendants responsible for your burnout? Please explain in detail with example.<br>- Were the stringent rules and regulations of this health facility responsible for your burnout? Please explain in detail with example.
403	How does the burnout affect your work?	Probe:<br>- Your relationship with your senior/supervisor?<br>- Your relationship with your co-workers?<br>Your relationship with your patients or their attendants?
404	How do you cope when you are in the state of burnout?	Please explain in detail with example.
405	What do you suggest how can we mitigate the burnout from maternity staff?	Please explain in detail with example.
**Q5**	Theme: Relationship with co-workers	
	Now I would like to ask about your relationship with your co-workers and supervisors	
501	The challenges you just mentioned, what kind of support do you receive from your co-workers AND supervisors to deal with these challenges?	Ask separately about co-workers and supervisors
502	In general, how would you describe your relationship with your co-workers	Why do you think the relationship is good, ok, or bad
503	In general, how would you describe your relationship with your supervisors	Why do you think the relationship is good, ok, or bad
504	What do you do to make this relationship supportive?	How do you do?<br>Why do you do?
505	What do you expect from co-workers to improve the relationship?	Probe: why do you such expectations
506	What do you expect you're your supervisor to improve the relationship?	Probe: why do you such expectations
	**Sub-theme: suggestions and recommendations**	

407	In your opinion, how can the relationship with co-workers be strengthened or more supportive?	Why do think this strengthen the relationship?
408	In your opinion, how can the relationship with supervisors be strengthened or more supportive?	Why do think this strengthen the relationship?
**Q5**	Theme: Provider interaction with patients	
	Now I would like to ask about patient-provider interaction in this health facility	
	**Sub-theme: Care for women with varying needs**	
501	Can you please describe what happens in a typical case of a delivery?<br>Encourage the participant to develop a flow diagram of the activities?<br>**FLOW DIAGRAM.**	Ask respondents to describe typical journey of a women who comes to deliver the baby at the health facility and then leaves the facility after getting discharged. For example, before delivery in ward, labour room, post-delivery?<br><br>Who deals with women at every stage?<br>How much time do you spend on each phase of care?<br>What kind of care support is provided?<br>How long does a woman spend at every stage?<br>How is it ensure that best care is provided to the woman?
502	Can you describe in details what kind of care is provided to women at each stage?	
503	In your opinion, do all women have similar or different expectations and needs? Can you describe in detail about these differential expectations and needs of women?	Ask separately about physical care and emotional care
504	How do you identify different needs of women?	Personal issues, psychological distress, functional disability, illiteracy, language
505	How do you meet these differential needs and expectations? Can you give examples?	Does someone provide informational support to women?<br>Does someone provide emotional and psychological support?
	**Sub-theme: Challenges in meeting patients' needs**	
506	What are the major challenges you face meeting these varying needs/expectation of women?	What type of difficulties you face providing information?<br>What type of difficulties you face while providing emotional and psychological support?
507	What happens if you fail to meet those expectations and needs?	
	I will now particularly ask about different types of patients that may come to you for childbirth, and would like you to explain how do meet the differential needs to these patients?	
508	How would you deal with a woman who cannot see?	What difficulties do you face dealing with such women?<br>What trainings have you received to deal with such situations?

509	How would you deal with a woman who is physically disabled?	What difficulties do you face dealing with such women?<br>What trainings have you received to deal with such situations?
510	How would you deal with a woman who is scared, anxious and constantly crying?	What difficulties do you face dealing with such women?<br>Have you received any trainings to deal with such situations?
511	How do you deal with woman who cannot understand your language?	What difficulties do you face dealing with such women?<br>Have you received any trainings to deal with such situations?
512	What should be done to deal with these challenges?	Why? How could this implemented?
**Q6**	**Theme: Mistreatment**	
	Now I would like to ask you about patient-provider relationship	
601	Tell me about particular challenging interactions with a women experienced by your colleague. It can be even based on your own experience.	Probes: Do you ever have disagreements with clients?<br>What kind of disagreements?<br>How are they resolved?<br>What hinders provider relationship with clients?<br>Have you been trained on dealing with such situation?
602	Some people say that in public health facilities the behaviour of service providers toward women are is not good? What are your views on that?	Why do you think this way?<br>Have you ever experienced or witnessed tension between care provider and patients?<br>Why was that? How it was dealt?
603	Few people handle the most difficult situation/patient very calmly while ensuring respect and dignity of patient? How do you think they manage it and what is the reason?	
604	Can you suggest changes should be made in this health facility to ensure that varying needs of all women are adequately addressed during intrapartum care?	Instructions: Please ask separately about: capacity building of health facility staff, work ethics, team bonding, institutional policies, and supervision.<br>Probes:<br>- Do you think building capacity of staff could help meeting these needs? Why do you think you? And how this could be done?<br>- Do you think improving work environment for staff could help meeting these needs? Why do you think you? And how this could be done?<br>- Do you think supportive supervision and monitoring could meeting these needs? Why do you think you? And how this could be done?
	**Thank you for your responses. Now I will give you some hypothetical scenarios and will ask you if you were in the given situation how would you deal with the situation.**	
605	A woman who followed a normal pregnancy over nine months has come to the hospital for childbirth. In view	In your current settings, how would you deal with such situation? Why?

	of possible serious complications, the doctor advised the woman to undergo C-section. The woman has refused to undergo the procedure since it was a normal pregnancy and her family members are not in favour of operation. How would you handle the situation?	
606	Due to prolonged labour women has been shouting with severe pain. Despite repeated explanations, the woman is not adhering to the instructions and keep asking for operations. The doctor used harsh tone to deal with her but eventually has slapped the woman.	What are your views about such attitude of doctor? Why do you feel this?<br>Under what circumstances do you think being verbal harsh or handling physically is acceptable?
607	A woman has recently given birth and needs stitching; another women in the final stage of labour needs to be moved to labour room for delivery. However, the anaesthesia is not available. The provider has following choices: a) perform stiches without anaesthesia and free up the bed to other women can come in; b) take a risk and wait for the anaesthesia in this case the other woman may deliver in the ward.	What will be the role of providers in such situation?<br>How would you deal such situation and why?
608	A nurse assisting the doctor has noticed that the doctor is performing post-delivery stitching without anaesthesia and the women is screaming due to pain and complaining not do it. Stopping the doctor may lead to negative consequences for the nurse. What would you if you were that nurse?	How would you deal such situation and why?
609	A woman with normal pregnancy/labour, constantly screaming due to labour pain and asking for pain relief medication. But, the physician remained busy with an emergency in the labour room	How would you deal with such situation and Why?
610	During childbirth, a woman is very restless, not responding to the instructions, and persistently asking for a companion. The person who has accompanied is her husband. Having a companion in labour room is not permissible according to the policy and other women	How would you deal such situation and why?

	are also resisting to due confidentiality issue. How would you deal with the situation?	
611	A 35-year-old anaemic woman after giving birth to a 12th child expressed the desire for tubal ligation, and requested not to inform her family members, as they often physically abuse her, and her husband desires for more children. As per law, the service provider needs to have signed consent from her husband.	How would you deal such situation and why?
612	A 20-year woman giving birth for the first time. Knowing that she is suffering from depression and is very scared of medical procedures, the doctor decides not to share details like what to expect from labour or childbirth and the operative procedure to be used for inducing labour and childbirth. The doctor thinks that it will further disturb her.	What are your views about the decision of the doctor?<br>Under what circumstances do you think such information should not be shared with the women to avoid unrest or panic among women?
**7**	**Sub-theme: Suggestions and recommendation**	
701	What do you suggest which types of initiatives can be taken to improve the relationship between providers and patients? Who and how these initiatives should be taken? If someone asked you what would be your suggestions?	Ask separately about:<br>• Capacity building of health facility staff<br>• Work ethics<br>• Team bonding<br>• Institutional policies<br>• Supervision etc.<br>• Why do you feel this?<br>• How these changes could be incorporated?
702	Anything else that you would like to suggest to improve the maternity care in this hospital?	

Supplement to: Asim M, Hameed W, Khan B, Saleem S, Avan BI. Applying the COM-B model to understand the drivers of mistreatment during childbirth: a qualitative enquiry among maternity care staff. Glob Health Sci Pract. 2022;10(6):e2200267. https://doi.org/10.9745/GHSP-D-22-00267

تفصیلی انٹرویو گائیڈ برائے میٹرنٹی عملہ

تفصیلی انٹرویو کا سوالنامہ مرکزِ صحت کے عملے کے لیے:

نمبر	سوال	جواب
**بنیادی معلومات:**		
101	انٹرویو کی تاریخ	|___|___| / |___|___| / |___|___|
102	ضلع کا نام	۱۔ٹھٹہ ۲۔سجاول
103	تعلقہ کا نام	
104	یونین کانسل کا نام	
105	مرکزِ صحت کا نام	
106	انٹرویور کا نام	
107	جواب دہندہ کی قسم	1 منیجمنٹ کا عملہ<br>2 طبّی عملہ<br>3 معاونتی عملہ
108	جنس	۱۔مرد ۲۔عورت
109	عمر (سالوں میں)	|___|___|سال
110	اس مرکزِ صحت پر آپ کا عہدہ کیا ہے؟	۱۔میڈیکل سپریڈنٹنٹ ۲۔انچارج—زچہ وبچہ شعبہ<br>۳۔میڈیکل آفیسر ۴۔نرس<br>۵۔مڈوائف ۶۔ماسی<br>۷۔چوکیدار/گارڈ<br>دیگر وضاحت کریں________________
111	آپ کو اس مرکز میں کام کرتے ہوئے کتنا عرصہ ہو گیا ہے؟	سال |___|___|
112	آپ کا پیشہ ورانہ مجموعی تجربہ کتنا ہے؟	سال |___|___|
113	آپ کی تعلیمی قابلیت کتنی ہے؟	
114	آپ کس شفٹ میں کام کرتی/کرتے ہیں؟	۱۔صبح ۲۔شام<br>۳۔رات دیگر وضاحت کریں________________
115	انٹرویو شروع ہونے کا وقت	|___|___|:|___|___|
116	انٹرویو ختم ہونے کا وقت	|___|___|:|___|___|

Supplement to: Asim M, Hameed W, Khan B, Saleem S, Avan BI. Applying the COM-B model to understand the drivers of mistreatment during childbirth: a qualitative enquiry among maternity care staff. Glob Health Sci Pract. 2022;10(6):e2200267. https://doi.org/10.9745/GHSP-D-22-00267

نمبر	سوالات	کریدیں
Q1	**سب سے پہلے میں آپ سے اس مرکزِ صحت کے بارے میں چند سوالات کرنا چاہوں گی/گا مثلاً شعبہ زچہ وبچہ کا بنیادی ڈھانچہ، عملہ، فراہم کی جانے والے سہولیات کی اقسام، مریضوں کی تعداد، دستیاب رہنما اصول/ ہدایاتی، اور یہ کہ اس مرکزِ صحت میں روزمرہ کس طرح کام انجام دیا جاتا ہے اور انتظامات کس طرح سنبھالے جاتے ہیں۔**	
	مرکزی موضوع: مرکزِ صحت کا ڈھانچہ اور معمول کے کام:	
101	اس زچہ وبچہ کے شعبہ میں ماں اور بچے کے لیے کس قسم کی سہولیات فراہم کی جاتی ہیں؟	**ہدایات:** ماں اور بچے کی سہولیات کے بارے میں علیحدہ علیحدہ پوچھیں
102	اس زچہ وبچہ کے شعبہ میں کتنے کمرے اور بستر دستیاب ہیں؟<br>یہاں مہینے میں اوسطاً کتنے بچوں کی پیدائش ہوتی ہے؟	**علیحدہ علیحدہ پوچھیں:**<br>- ایک ماہ میں نارمل پیدائش کتنی ہوتی ہیں؟<br>- ایک ماہ میں آپریشن کے ذریعے کتنی پیدائش ہوتی ہیں؟<br>- کون سے دن آپریشن کے لیے مخصوص ہیں؟<br>- عموماً مہینے کے کون سے دنوں میں زچگی بہت زیادہ یا بہت کم ہوتی ہے؟
103	کیا آپ تفصیلاً بتائینگی یہاں کا عملہ کن لوگوں پر مشتمل ہے اور اس کی درجہ بندی کس طرح سے ہے؟<br>**برائے مہربانی آرگینوگرام / یا خاکہ بنا کر بتایئے۔**	**علیحدہ پوچھیں:**<br>- مجموعی طور پر عملے کی تعداد کتنی ہے؟<br>- ہر درجہ پر سپروائیزر کون ہے؟<br>- کام کی شفٹ کس طرح بنائی جاتی ہے؟ مثلاً صبح، شام اور رات کے اوقات کار اور کام کرنے کا عملہ<br>- ہر درجہ پر فیصلہ سازی کا اختیار کس کے پاس ہے؟ اور وہ شخص کس قسم کی فیصلہ سازی کر سکتا ہے؟ کچھ فیصلوں کی مثالیں دیجیے۔
104	گزشتہ ۲ سالوں میں کس قسم کی طبّی اور غیر طبّی ٹریننگ یہاں کے عملے کو دی گئی؟	**کریدیں:**<br>- کیا آپ مجھے ہر ٹریننگ کے مقاصد بتا سکتی ہیں؟<br>- کس کی طرف سے یہ ٹریننگ فراہم کی گیٔ؟<br>- کتنی مرتبہ یہ ٹریننگ منعقد کی گئیں اور کس عملے کے لیے یہ منعقد کی گئیں؟<br>- ان ٹریننگ کا دورانیہ کتنا تھا؟
105	کیا آپ تفصیلاً بیان کر سکتی ہیں کہ مریضوں کی معلومات اکٹھا کرنا اور اس کو محفوظ کرنے کا طریقہ کار کیا ہے؟	**کریدیں:**<br>- برائے مہربانی تفصیلاً بتایئے کہ خواتین کے ہسپتال میں داخلے سے لے کر چھٹی ملنے تک ہر مرحلے پر کس قسم کی معلومات اکٹھا کی جاتی ہیں؟<br>- کیا آپ بتا سکتی ہیں کہ ہر مرحلے پر کس قسم کے فارم/ریجسٹر کو پُر کرنا ہوتا ہے اور اس کو بھرنے کے ذمہ داری کس کی ہوتی ہے؟<br>- خواتین کے فارم/فائل کہاں محفوظ کیے جاتے ہیں اور ان کو کس طرح ضرورت پڑنے پر کس طرح نکالا جاتا ہے؟

		- خواتین کی معلومات کو کسی کمپیوٹر میں اندراج کیا جاتا ہے؟ اگر ہاں، تو کس قسم کی معلومات کا اندراج کمپیوٹر میں ہوتا ہے؟
106	کیا آپ مجھے بتائیں گی کہ خواتین کی معلومات کو فیصلہ سازی کے لیے کس طرح استعمال کیا جاتا ہے؟ ۱۔ فیصلہ سازی مریضوں کی ضروریات کو پورا کرنے کے لیے؛ ۲۔ اس زچہ و بچہ شعبہ کی مجموعی طور پر کارکردگی کو بہتر بنانے کے لیے یا سہولیات کے معیار کو بہتر بنانے کے لیے؟	**کریدیں:**<br>- کیا آپ مثال دے کر بتا سکتی ہیں خواتین کی معلومات کو استعمال کرتے ہوئے اس کو مناسب اور ضرورت کے مطابق دیکھ بھال فراہم کی گئی؟<br>○ کون فیصلہ سازی کرتا ہے؟<br>○ فیصلہ سازی کا طریقہ کار کیا ہوتا ہے؟<br>- کیا آپ مثال دے کر بتا سکتی ہیں خواتین کی معلومات کو استعمال کرتے ہوئے اس مرکزِ صحت کی کارکردگی کو بہتر بنایا گیا ہو؟<br>○ کون اس طرح کی فیصلہ سازی کرتا ہے؟<br>○ فیصلہ سازی کا طریقہ کار کیا ہوتا ہے؟
	سہولیات کے معیار کی یقین دہانی	
107	**اب میں آپ سے ان طریقہ کار یا نظام کے بارے میں پوچھنا چاہوں گی/گا جو مریضوں کو فراہم کی جانے والی سہولیات کے معیار کو بہتر بنانے کے لیے بنائے گئے ہیں۔ اس بات کی یقین دہانی کرواتی/کرواتا جاؤں کے ہم خاص طور پر ان سہولیات کے بارے میں جاننا چاہیں گے جو زچگی کی خواتین کو فراہم کی جاتی ہیں۔**	
108	سہولیات فراہم کرنے کے لیے کون کون سی رہنما اصول/گائیڈ لائینز موجود ہیں؟	**کریدیں:**<br>طبّی سہولیات (مثلاً سرجری کرنا وغیرہ) اور غیر طبّی سہولیات (بات چیت کا طریقہ کار، مریضوں کا احترام یا معاونت کرنا وغیرہ)، یا مریضوں کا ریکارڈ رکھنے کے حوالے سے پوچھیں۔
109	اس بات کی یقین دہانی کس طرح کی جاتی ہے کہ زچگی کی سہولیات تعین کردہ رہنما اصولوں یا گائیڈ لائینز کے مطابق دی جائے؟	**کریدیں:**<br>**طبّی سہولیات کے حوالے سے:**<br>- کیا آپ کو تعین کردہ رہنما اصولوں کے پر کوئی تربیت دی گئی ہے؟<br>- ہر عملے کو تعین کردہ اصولوں پر عمل درآمد کروانا کس کی ذمہ داری ہے؟<br>- عملہ ان اصولوں پر عمل کرتا ہے یا نہیں کرتا، اس کی نگرانی کس طرح کی جاتی ہے؟<br>- کتنی مرتبہ کی عملے کی نگرانی کی جاتی ہے؟<br>- اگر کوئی تعین کردہ اصولوں پر عمل درآمد نہ کرے تو کس طرح کے اقدامات کئے جاتے ہیں؟<br>- کوئی اس طرح کا فورم ہے جہاں سہولیات کے معیار میں بہتری کے لیے نظر ثانی کی جاتی ہو (مثلاً ماہانہ میٹنگ وغیرہ)۔ اس کے بارے میں بتائیے گا۔<br>**غیر طبّی دیکھ بھال:**<br>- کیا کوئی تعین کردہ رہنما اصول ہیں جو یہ بتائیں کہ عملے کو مریضوں کے ساتھ کس طرح کا رویہ رکھنا چاہیے؟ (مثلاً احترام، دہستانہ، موثر بات چیت وغیرہ)

		- اس بات کو کس طرح یقینی بنایا جاتا ہے کہ عملے کا مریضوں کے ساتھ رویہ اچھا ہو۔<br>- اگر کوئی تعین کردہ اصولوں پر عمل درآمد نہ کرے تو کس طرح کے اقدامات کئے جاتے ہیں؟
110	ڈسٹرک ہیلتھ آفیسر اس مرکزِ صحت میں فراہم کی جانے والی زچگی کی سہولیات کو معیاری بنانے کے لیے کیا کردار ہے؟	- ان کی طرف سے کس قسم کی نگرانی یا تعاون کیا جاتا ہے؟<br>- ڈسٹرک کی طرف سے نگرانی یا معاونت کون کرتا ہے؟<br>- ڈسٹرک کی طرف سے نگرانی یا معاونت سہولیات کے معیار کو بہتر بنا کس حد تک موثر ہوتی ہے؟
Q2	موضوع: اہم تصورات کے حوالے سے سمجھ بوجھ	
	اب میں کچھ اہم تصورات کے حوالے سے آپ کی سمجھ بوجھ جاننا چاہوں گا جو صحت کے حوالے سے جن کا استعمال ہوتا ہے۔ میں آپ کو ایک تصویر دیکھاؤں گی/گا اور کچھ الفاظ آپ کے سامنے پڑھوں گی، آپ سے کہوں گی/گا کہ آپ اس کو اپنی سمجھ کے مطابق بیان کریں۔ میں آپ کو دوبارہ یاد دہانی کروادوں کہ اس میں کچھ صحیح یا غلط کی بات نہیں ہو رہی ہے۔ ہم صرف آپ کی رائے جاننا چاہتے ہیں۔	
201	ہدایت: برائے مہربانی اس تصویر کو جواب دہندہ کو دکھائیں اور دیے گئے سوالات پوچھیں:<br>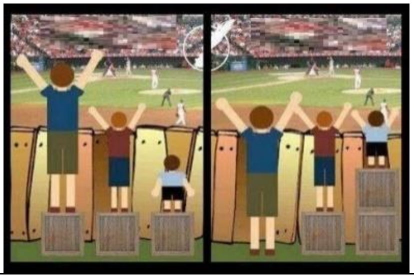	- کیا آپ بیان کر سکتے ہیں آپ کو اس تصویر میں کیا دکھایا گیا ہے؟<br>- کیا آپ اپنی زورمرہ کی زندگی سے کوئی مثال دے کر اس تصویر کو بیان کر سکتی ہیں؟<br>- آپ اس تصویر کو اس مرکز صحت میں فراہم کی جانے والی زچہ و بچہ کی سہولیات سے منسلق کر سکتی ہیں؟<br>- آپ کی رائے میں اس مرکز صحت پر زچگی کے لیے آنے والی خواتین کی ضروریات مختلف ہوتی ہیں؟<br>- آپ ہر خاتون کی مختلف ضرورت کو کس طرح پورا کرتی ہیں؟<br>- آپ کو ان کی مختلف ضروریات کو پورا کرنے میں کن دشواریوں کو سامنا ہوتا ہے؟
202	آپ کے مطابق لفظ 'احترام' سے کیا مراد ہے؟	- کیا آپ اپنی زورمرہ کی زندگی سے کوئی مثال دے کر اس لفظ کو بیان کر سکتی ہیں؟<br>- آپ اس لفظ کو اس مرکز صحت میں فراہم کی جانے والی زچہ و بچہ کی سہولیات سے منسلق کر سکتی ہیں؟<br>- آپ اس بات کی یقین دہانی کیسے کرتی ہیں کہ زچگی کی سہولیات احترام کے ساتھ فراہم کی جائیں۔<br>- اس بات کی یقین دہانی کہ زچگی کی خواتین کے ساتھ عزت و احترام کا رویہ رکھا جائے، آپ کو کن دشواریوں کو سامنا ہوتا ہے؟
203	آپ کے مطابق لفظ "تعاون"/معاونت/مدد سے کیا مراد ہے؟	- کیا آپ اپنی زورمرہ کی زندگی سے کوئی مثال دے کر اس لفظ کو بیان کر سکتی ہیں؟<br>- آپ اپنی فیملی (بیوی/شوہر، بچے، ماں وغیرہ) کی کس طرح مدد/معاونت کرتے ہیں؟<br>- آپ اس لفظ کو اس مرکز صحت میں فراہم کی جانے والی زچہ و بچہ کی سہولیات سے منسلق کر سکتی ہیں؟<br>- زچگی کی خواتین کے ساتھ کس قسم کی معاونت/مدد کی جاتی ہے؟ کیا آپ کچھ مثالیں دی سکتی ہیں؟
204	آپ کے مطابق لفظ "صحت کی دیکھ بھال میں اخلاقیات" سے کیا مراد ہے؟	- کیا آپ کچھ مثالوں کی مدد سے اس کو بیان کر سکتی ہیں؟<br>- آپ اس لفظ کو اس مرکز صحت میں فراہم کی جانے والی زچہ و بچہ کی سہولیات سے منسلق کر سکتی ہیں؟ کیا آپ کچھ مثالیں دی سکتی ہیں؟

205	آپ کے مطابق لفظ ‘مریضوں کے حقوق" سے کیا مراد ہے؟	- کیاآپ کچھ مثالوں کی مدد سے اس کو بیان کرسکتی ہیں؟<br>- آپ اس لفظ کو اس مرکز صحت میں فراہم کی جانے والی زچہ وبچہ کی سہولیات سے منسلق کرسکتی ہیں؟ کیاآپ کچھ مثالیں دی سکتی ہیں؟<br>- آپ اس بات کی یقین دہانی کیسے کرتی ہیں کہ زچگی کی سہولیات فراہم کرنے کے دوران مریضوں کے حقوق کاخیال رکھاجائے؟<br>- زچگی کی سہولیات فراہم کرنے کے دوران مریضوں کے حقوق پورے کرنے کے لیے آپ کو کن دشواریوں کو سامناہوتاہے؟
206	آپ کے خیال میں خواتین صحت کی سہولیات کے حوالے سے کون کون سے حقوق میں جو اس مرکز صحت پر زچگی کروانے آتی ہیں؟	کریدنے کے لیے کچھ حقوق مندرجہ ذیل ہیں:<br>- معلومات کاحق (موثر بات چیت)<br>- رضامندی (مریض سے جسمانی معائنہ سے پہلے رضامندی لینا)<br>- خودمختاری (مریض کی مرضی کااحترام کرنا)<br>- رازداری<br>- بدکلامی نہ کرنا (مریض پر چیخنا، چلانہ، دھمکانہ وغیرہ)<br>- جسمانی تشدد نہ کرنا (مریض کو چانٹا مارنا وغیرہ)<br>- جنسی تشدد نہ کرنا<br>- تاخیر یا نظرانداز یا تنہانہ چھوڑنا<br>- غیرامتیازی سلوک<br>- معاونت/مدد
Q3	موضوع: عملے کاکرداراور ذمہ داریاں	
	**اب میں آپ سے اس مرکزِ صحت میں آپ کاکرداراور ذمہ داریوں کے بارے میں پوچھنا چاہوں گی۔**	
301	آپ کی اس مرکزِ صحت میں کیا ذمہ داریاں ہیں؟	- سرکاری طور پر آپ کی کیا ذمہ داریاں ہیں۔<br>- اپنی سرکاری ذمہ داریوں کے علاوہ کیاآپ کوئی اضافی کام کرتی ہیں؟
302	عموماً آپ کادن اس صحت کے مرکز میں کیسے گزرتا ہے؟ کیاآپ تفصیلاً بیان کریں گی؟<br>جوابدہندہ کی حوصلہ افزائی کریں کے وہ وقت کے حساب سے ایک خاکہ بنائے۔<br>TIMELINE	**ہدایت: برائے مہربانے جواب دہندہ سے کہیں کو وہ اپنے ہر کام کے بارے میں بتائیں—اس مرکز میں داخل ہونے سے لے کر گھر واپس جاتے تک۔**
303	آپ کا اپنے کام کے بارے میں کیا اچھا لگتا ہے؟	آپ کو اپنے کام میں یہ چیزیں کیوں پسند ہیں۔

Supplement to: Asim M, Hameed W, Khan B, Saleem S, Avan BI. Applying the COM-B model to understand the drivers of mistreatment during childbirth: a qualitative enquiry among maternity care staff. Glob Health Sci Pract. 2022;10(6):e2200267. https://doi.org/10.9745/GHSP-D-22-00267

	وہ کون سے چیزیں ہیں جو آپ کے لیے حوصلہ افزاء ہیں؟	
304	آپ کو اپنے کام کے حوالے سے کون سی چیز سب سے کم پسند ہے یا اچھی نہیں لگتی؟	آپ کو اپنے کام میں یہ چیزیں کیوں پسند نہیں ہیں۔
305	آپ کو اپنے روزمرہ کی کام میں کن مشکلات کا سامنا کرنا پڑتا ہے؟<br>سب سے پہلے، کیا آپ مجھے یہ بتا سکتی ہیں کہ آپ کو اپنے ساتھیوں کے حوالے سے کن مشکلات کا سامنا کرنا پڑتا ہے؟	**کریدیں:**<br>- آپ کو کیوں لگتا ہے کہ یہ آپ کے لیے مشکلات ہے؟<br>- آپ کے خیال میں آپ کو ان مشکلات کا سامنا کیوں کرنا پڑتا ہے؟<br>- آپ ان مشکلات سے کس طرح نمٹتی ہیں؟<br>- اس مرکزِ صحت پر اس قسم کی مشکلات سے نمٹنے کے لیے کوئی واضح طریقہ کار یا رہنما اصول ہیں؟<br>- یہ طریقہ کار یا رہنما اصول ان مشکلات کو حل کرنے میں کس حد تک موثر ہیں۔<br>- عموماً آپ کے اپنے ساتھی ورکر کے ساتھ کیسے تعلقات ہیں۔<br>- آپ کے خیال میں اپنے ساتھی ورکر کے ساتھ تعلقات کو کس طرح بہتر بنایا جا سکتا ہے؟
306	کیا آپ مجھے یہ بتا سکتی ہیں کہ آپ کو اپنے سپروائزر یا سینئر کے حوالے سے کن مشکلات کا سامنا کرنا پڑتا ہے؟	**کریدیں:**<br>- آپ کو کیوں لگتا ہے کہ یہ آپ کے لیے مشکلات ہے؟<br>- آپ کے خیال میں آپ کو ان مشکلات کا سامنا کیوں کرنا پڑتا ہے؟<br>- آپ ان مشکلات سے کس طرح نمٹتی ہیں؟<br>- اس مرکزِ صحت پر اس قسم کی مشکلات سے نمٹنے کے لیے کوئی واضح طریقہ کار یا رہنما اصول ہیں؟<br>- یہ طریقہ کار یا رہنما اصول ان مشکلات کو حل کرنے میں کس حد تک موثر ہیں۔<br>- عموماً آپ کے اپنے سپروائزر یا سینئر کے ساتھ کیسے تعلقات ہیں۔<br>- آپ کے خیال میں اپنے سپروائزر یا سینئر کے ساتھ تعلقات کو کس طرح بہتر بنایا جا سکتا ہے؟
307	کیا آپ مجھے یہ بتا سکتی ہیں کہ آپ کو مریضوں کے حوالے سے کن مشکلات کا سامنا کرنا پڑتا ہے؟	**کریدیں:**<br>- آپ کو کیوں لگتا ہے کہ یہ آپ کے لیے مشکلات ہے؟<br>- آپ کے خیال میں آپ کو ان مشکلات کا سامنا کیوں کرنا پڑتا ہے؟<br>- آپ ان مشکلات سے کس طرح نمٹتی ہیں؟<br>- اس مرکزِ صحت پر اس قسم کی مشکلات سے نمٹنے کے لیے کوئی واضح طریقہ کار یا رہنما اصول ہیں؟<br>- یہ طریقہ کار یا رہنما اصول ان مشکلات کو حل کرنے میں کس حد تک موثر ہیں۔

308	کیاآپ مجھے یہ بتاسکتی ہیں کہ آپ کو مریضوں کے ساتھ آنے والے لوگوں کے حوالے سے کن مشکلات کا سامنا کرناپڑتاہے؟	**کریدیں:**<br>- آپ کو کیوں لگتاہے کہ یہ آپ کے لیے مشکلات ہے؟<br>- آپ کے خیال میں آپ کوان مشکلات کا سامنا کیوں کرناپڑتاہے؟<br>- آپ ان مشکلات سے کس طرح نمٹتی ہیں؟<br>- اس مرکزِ صحت پراس قسم کی مشکلات سے نمٹنے کے لیے کوئی واضح طریقہ کاریار ہنمااصول ہیں؟<br>- یہ طریقہ کاریار ہنمااصول ان مشکلات کو حل کرنے میں کس حدتک موثرہیں۔
309	کیاآپ مجھے یہ بتاسکتی ہیں کہ آپ کواس مرکز صحت کے قائدے اور قوانیں (مثلاًریکارڈرکھنا،آفس کے اوقات وغیرہ) کے حوالے سے کن مشکلات کا سامنا کرناپڑتاہے؟	**کریدیں:**<br>- آپ کو کیوں لگتاہے کہ یہ آپ کے لیے مشکلات ہے؟<br>- آپ کے خیال میں آپ کوان مشکلات کا سامنا کیوں کرناپڑتاہے؟<br>- آپ ان مشکلات سے کس طرح نمٹتی ہیں؟<br>- اس مرکزِ صحت پراس قسم کی مشکلات سے نمٹنے کے لیے کوئی واضح طریقہ کاریار ہنمااصول ہیں؟<br>- یہ طریقہ کاریار ہنمااصول ان مشکلات کو حل کرنے میں کس حدتک موثرہیں۔
4	موضوع:پروائیڈرکابرن آئوٹ ہونا	
401	عموماًکام کی زیادتی یاکام کی نوعیت، کی وجہ سے ہم جسمانی یاذہنی طورپر نڈھال ہوجاتے ہیں۔ جسکی وجہ سے ہماری کام میں دلچپسی کم ہوجاتی ہے اور ہمیں کبھی لگتاہے کہ ہمارے کام کرنے کی صلاحیت میں کمی آئی ہے۔<br>اس تصورکو ہم انگریزی میں Burnout کہتے ہیں۔<br><br>کیاآپ کے ساتھ کبھی ایساہواہے؟	
402	ایساآپ کے ساتھ کب ہوا؟<br>آپ کے خیال میں اس کی وجہ کیا تھی؟	**کریدیں:**<br>کیاسپروائزیاسینئرآپ کے لیے **Burnout** کی وجہ بنی۔<br>کیاآپ کے ساتھی آپ کے لیے **Burnout** کی وجہ بنی۔<br>کیامریض یاان کے ساتھ موجودلوگ آپ کے لیے **Burnout** کی وجہ بنے۔<br>کیاادارے کے قانون یاقائدے آپ کے لیے **Burnout** کی وجہ بنے۔

403	Burnoutآپ کے کام پر کس طرح اثر انداز ہوا۔	**کریدیں:**<br>آپ اپنے سپر وائزر یا سینئر کے ساتھ تعلق پر **Burnout** کیسے اثر انداز ہوا۔<br>آپ اپنے ساتھی ورکر کے ساتھ تعلق پر **Burnout** کیسے اثر انداز ہوا۔<br>مریض یا ان کے ساتھ موجود لوگ کے ساتھ تعلق پر **Burnout** کیسے اثر انداز ہوا۔
404	جب Burnoutہوتا ہے تو آپ اس سے کس طرح نمٹتی ہیں۔	کیا آپ مثال دی کر بتا سکتی ہیں؟
405	زچہ و بچہ کے عملے کو Burnoutسے بچانے کے لیے آپ کیا تجاویز دیں گی۔	آپ کو ایسا کیوں لگتا ہے؟
Q5	موضوع: پر وائیڈر کے مریضوں کے ساتھ تعلقات	
	**اب میں آپ سے پر وائیڈر اور مریضوں کے درمیان تعلقات**	
	**Sub-theme: Care for women with varying needs**	
501	کیا آپ تفصیلاً بتائینگی کی عام طور پر بچے کی پیدائش کن مراحلے سے گزرتے ہوئے ہوتی ہے؟<br>جوابدہندہ کی حوصلہ افزائی کریں کہ وہ ہر مرحلے کو کاغذ پر نقش کر کے بتائے؟<br>FLOW DIAGRAM	جواب دہندہ سے کہیں کہ وہ خواتین کا سفر تفصیلی بیان کریں۔اس کے مرکز میں آنے سے لے کر ہسپتال سے چھٹی ملنے تک وہ کن مراحل سے گزرتی ہے–مثلاً زچگی کے پہلے، زچگی کے دوران، اور زچگی کی بعد۔<br>- ہر مرحلے پر کس قسم کی دیکھ بھال فراہم کی جاتی ہے؟<br>- ہر مرحلے پر خاتون کو کتنا وقت لگتا ہے؟<br>- اس بات کی یقین دہانی کہ خاتون کو بہترین دیکھ بھال ملے؟
502	ہر مرحلے پر خواتین کو کس قسم کی مدد فراہم کی جاتی ہے؟	
503	آپ کے خیال میں کیا تمام خواتین کی ایک جیسی یا مختلف توقعات یا ضروریات ہوتی ہیں؟<br>کیا آپ بتائینگی کی ان کی کس قسم کی مختلف توقعات اور ضروریات ہوتی ہیں؟	**کریدیں:**<br>جسمانی طور پر اضافی ضروریات اور ذہنی طور پر اضافی ضروریات کے بارے میں علیحدہ علیحدہ پوچھیں۔
504	آپ خواتین کی مختلف ضروریات کی کس طرح نشاندہی کرتے ہیں اور اس کو کس طرح پورا کرتے ہیں؟	- ذہنی دباؤ کا شکار خواتین، معذوری، ناخواندگی، زبان سے واقفیت کا نہ ہونا
505	خواتین کی مختلف توقعات یا ضروریات کو کس طرح پورا کیا جاتا ہے؟ کیا آپ مثال دی کر بتا سکتی ہیں؟	کیا ان کو کوئی معلومات فراہم کی جاتی ہے<br>ان کو جذباتی یا سماجی (نفسیاتی) مدد دی جاتی ہے

	**Sub-theme: Challenges in meeting patients' needs**	
ان کو معلومات فراہم کرنے میں کیا مشکلات ہوتی ہیں<br>ان کو جذباتی یا سماجی (نفسیاتی) مدد دینے میں کیا مشکلات ہوتی ہیں	آپ کو خواتین کی مختلف توقعات یا ضروریات کو پورا کرنے میں کن مشکلات کا سامنا کرنا پڑتا ہے؟	506
	اگر آپ خواتین کی مختلف توقعات یا ضروریات کو پورا نہ کر سکیں تو کیا ہوتا ہے؟	507
**اب میں آپ کو کچھ مثالیں دوں گی/گا اور پوچھوں گا کہ آپ اس قسم کی مریضوں کی توقعات پر کیسے پورا اترتی ہیں یا ان کی اضافی ضروریات کو پورا کرتی ہیں؟**		
اس طرح کی خواتین کی دیکھ بھال فراہم کرنے میں کن مشکلات کا سامنا کرنا پڑتا ہے؟<br>اس طرح کی خواتین کی دیکھ بھال فراہم کرنے کے لیے کوئی تربیت آپ کو دی گئی ہے؟	آپ ان خواتین کی کس طرح دیکھ بھال کرتی ہیں جن کی بینائی نہیں ہوتی؟	508
اس طرح کی خواتین کی دیکھ بھال فراہم کرنے میں کن مشکلات کا سامنا کرنا پڑتا ہے؟<br>اس طرح کی خواتین کی دیکھ بھال فراہم کرنے کے لیے کوئی تربیت آپ کو دی گئی ہے؟	آپ ان خواتین کی کس طرح دیکھ بھال کرتی ہیں جن کو جسمانی معذوری ہے؟	509
اس طرح کی خواتین کی دیکھ بھال فراہم کرنے میں کن مشکلات کا سامنا کرنا پڑتا ہے؟<br>اس طرح کی خواتین کی دیکھ بھال فراہم کرنے کے لیے کوئی تربیت آپ کو دی گئی ہے؟	آپ ان خواتین کی کس طرح دیکھ بھال کریں گی جو بہت ہی ڈری ہوئی ہوں، یا بے چین یا گھبرائی ہوئی ہوں یا مستقل رو رہی ہوں؟	510
اس طرح کی خواتین کی دیکھ بھال فراہم کرنے میں کن مشکلات کا سامنا کرنا پڑتا ہے؟<br>اس طرح کی خواتین کی دیکھ بھال فراہم کرنے کے لیے کوئی تربیت آپ کو دی گئی ہے؟	آپ ان خواتین کی کس طرح دیکھ بھال کریں گی جو آپ کی زبان نہ جانتی ہو؟	511
آپ کو کیوں لگتا ہے کہ یہ اقدامات موثر ہوں گے؟<br>ان اقدامات کو اس مرکزِ صحت میں کس طرح لاگو کیا جاسکتا ہے؟	ان مشکلات سے نمٹنے کے لیے کس قسم کی اقدامات کرنے چاہیے؟	512
	بدسلوکی	Q6
	اب میں آپ کے پروائیڈر اور مریضوں کے تعلقات کے بارے میں جاننا چاہوں گی/گا۔	
کیا آپ کے کبھی مریضوں کے ساتھ اختلافات ہوئے ہیں؟<br>کس قسم کے اختلافات؟<br>ان کو کس طرح حل کیا جاتا ہے؟<br>مریض اور دیکھ بھال فراہم کرنے والوں کے درمیان اچھے تعلق بنانے میں کونسی رکاوٹیں ہوتی ہیں؟<br>کیا آپ کو اس قسم کی مشکل صورت حال سے نمٹنے کے لیے کوئی تربیت ملی ہے؟	مجھے اپنا یا اپنے کسی ساتھی کا کوئی ایسا تجربہ بتائیں جس میں حاملہ خواتین کو زچگی کی سہولیات فراہم کرنے میں بہت مشکلات کا سامنا کرنا پڑا ہو۔	601

602	کہاجاتاہے کہ سرکاری ہسپتالوں میں عملے کارویہ مریضوں کے ساتھ اچھانہیں ہوتا۔اس بارے میں آپ کی کیارائے ہے؟	آپ کوایسا کیوں لگتا ہے؟<br>کیاآپ نے مریض اور پروائیڈر کے درمیان تناٶ دیکھا ہے؟<br>اس کی کیاوجہ تھی اوراس کو کس طرح حل کیا گیا؟
603	کچھ لوگ ایسے ہوتے ہیں جو کسی بھی مشکل صورت حال یامریض کو بہت ہی اطمینان کے ساتھ حل کر لیتے ہیں،اس بات کاخیال رکھتے ہوئے مریض کی ساتھ کسی قسم کی بدتمیزی نہ ہو؟آپ کے خیال میں وہ لوگ کس طرح یہ باآسانی کر لیتے ہیں اوراس کی کیاوجوہات ہوسکتی ہیں؟	
604	کیاآپ بتاسکتی ہیں کہ اس مرکزِ صحت میں کس قسم کی تبدیلیوں کی ضرورت ہے تاکہ زچگی کے لیے آنے والی خواتین کی مختلف توقعات اور ضروریات پر پورا اترا جاسکے؟	ہدایت: برائے مہربانی علیحدہ علیحدہ بتائیں: عملے کی تربیت؛کام کرنے کاطریقہ کار؛ٹیم کی درمیان بہتر ہم آہنگی؛ادارے کی پالیسی اور بہتر نگرانی۔ کریدیں:<br>- آپ کے خیال میں عملے کو کس قسم کی تربیت دینے کی ضرورت ہے؟آپ کوایسا کیوں لگتا ہے؟یہ کس طرح ممکن ہوسکتا ہے؟<br>- آپ کے خیال میں مریضوں کی توقعات پر پورا اترنے کے لیے کام کاماحول بہتری بنانے کی ضرورت ہے؟آپ کوایسا کیوں لگتا ہے؟یہ کس طرح ممکن ہوسکتا ہے؟<br>- آپ کے خیال میں مریضوں کی توقعات پر پورا اترنے کے لیے سہولیات فراہمی کی عمل کی نگرانی کی ضرورت ہے؟آپ کوایسا کیوں لگتا ہے؟یہ کس طرح ممکن ہوسکتا ہے؟
	**آپ کے جوابات کاشکریہ۔اب میں آپ کو کچھ فرضی خاکے بتائوں گی/گااور جاننا چاہوں گا کہ آپ اس صورت حال کو کس طرح حل کرتے۔**	
605	ایک خاتون ہسپتال میں آتی ہے جس کا۹ماہ تک نارمل حمل رہاہے۔اس کی حالت دیکھنے کے بعد ڈاکٹر کو کچھ پیچیدگی نظر آتی ہے اور وہ اس کو آپریشن کروانے کی تجویز دیتا ہے۔مگر خاتون منع کردیتی ہے کیوں کہ اس کو پتا ہے کہ حمل نارمل ہے اوراس کے گھر والے بھی آپریشن سے منع کردیتے ہیں۔اس صورت میں پروائیڈر کو کیاکرنا چاہیے؟	آپ اس صورت حال میں کیاکریں گی اور کیوں؟
606	طویل لیبر کی وجہ سے ایک خاتون درد سے چلا رہی ہے۔اس کو کئی بار سمجھایا گیا مگر وہ نہیں مانتی۔ڈاکٹر اس پر چلائی مگر پھر بھی اس نے بات نہیں مانی۔آخر کار ڈاکٹر نے اس کو ایک تھپڑ مارا۔	ڈاکٹر کے رویہ کے حوالے سے آپ کی رائے کیا ہے؟آپ کوایسا کیوں لگتا ہے۔<br>آپ کے خیال میں کن صورت حال میں خاتون سے سختی سے پیش آنا یاجسمانی طور پر اس کو سمبھالنا قبل قبول ہے؟اور کیوں؟
607	ایک خاتون کی ابھی زچگی ہوئی ہے اوراس کو ٹانکے لگنے ہیں۔ایک اور خاتون کے بچے کی پیدائش ہونے کے قریب ہے، جس کو فوراً لیبر کے کمرے میں جگہ چاہیے۔اور جب کے سن کرنے کی دواموجود نہیں	اس صورت میں پروائیڈر کو کیاکرنا چاہیے؟<br>آپ اس صورت حال میں کیاکریں گی اور کیوں؟

	ہے جو ٹانکوں سی پہلے لگائی جاتی ہے۔ پر وائیڈر کے پاس دو آپشن ہیں :a) بناء سن کیے ٹانکے لگا کر بستر دوسری خاتون کو دے دے ؛b) خطرہ مول لے سن کرنے والی دوائی آنے کا انتظار کرے جس صورت میں دوسری خاتون کی زچگی بھی ہو سکتی ہے۔	
آپ اس صورت حال میں کیا کریں گی اور کیوں ؟	ایک نرس، ڈاکٹر کے ساتھ ڈیوٹی پر ہے۔ وہ یہ دیکھتی ہے کہ ڈاکٹر نہیں بچے کی پیدائش کے بعد بغیر سن کیے خاتون کو ٹانکے لگا رہی ہے اور خاتون درد سے چلا رہی ہے۔ نرس کے پاس دو آپشن ہیں :a) ڈاکٹر کو بغیر دوا ٹانکے لگانے سے منع کرے جو اس کے لیے برا ہو سکتا ہے یا وہ خاموش رہے۔<br>اس صورت میں پر وائیڈر کو کیا کرنا چاہیے ؟	608
آپ اس صورت حال میں کیا کریں گی اور کیوں ؟	ایک نارمل حمل کی خاتون لیبر میں مسلسل چلا رہی ہے اور درد کم کرنے لیے دوا مانگ رہی ہے۔ مگر ڈاکٹر لیبر کے کمرے میں ایک ہنگامی کیس کو دیکھ رہا ہے۔ اس صورت میں پر وائیڈر کو کیا کرنا چاہیے ؟	609
آپ اس صورت حال میں کیا کریں گی اور کیوں ؟	زچگی کے دوران ایک خاتون بہت بے چین ہے اور دی گئی ہدایات پر بالکل بھی عمل نہیں کر ہی اور مسلسل کہہ رہی ہے کہ اس کے ساتھی کو اندر بلایا جائے۔ جو شخص اس کے ساتھ آیا ہے وہ اس کا شوہر ہے۔ قائدے کے مطابق ساتھی لیبر کے کمرے میں نہیں جا سکتا اور لیبر کے کمرے میں موجود دیگر خواتین منع کر رہی ہیں کیونکہ یہ ان کے لیے رازداری کا مسئلہ ہے۔ اس صورت میں پر وائیڈر کو کیا کرنا چاہیے ؟	610
آپ اس صورت حال میں کیا کریں گی اور کیوں ؟	ایک ۳۵ سالہ خاتون جس میں خون کی کمی ہے اپنے ۱۲ بچے کی پیدائش کے بعد ڈاکٹر سے کہتی ہے کہ اس کی نل بندی کر دی جائے۔ وہ یہ بھی بتاتی ہے کہ اس کے گھر والے اس پر تشدد کرتے ہیں اور ان کو مزید بچوں کی خواہش ہے۔ پالیسی کے مطابق پر وائیڈر کو شوہر سے اظہارِ رضامندی پر دستخط لینا لازمی ہے۔ اس کے بغیر وہ نل بندی نہیں کر سکتی۔	611
ڈاکٹر کے اس عمل کے حوالے سے آپ کی رائے کیا ہے ؟ آپ کو ایسا کیوں لگتا ہے ؟<br>آپ کے خیال میں کن صورت حال میں یہ بہتر ہے کہ خاتون کے ساتھ معلومات فراہم نہ کرنا قبل قبول ہے ؟ اور کیوں ؟	ایک ۲۰ سال کی خاتون پہلی مرتبہ زچگی کروا رہی ہیں اور وہ آپریشن یا سرجری سے بہت ڈری ہوئی ہے۔ ڈاکٹر یہ جانتے ہوئے کہ خاتون کو ڈپریشن ہے، یہ فیصلہ کرتا ہے کہ اس کو زچگی کے مرحلے اور اس کے طریقہ کار کے بارے میں نہ بتایا جائے تاکہ اس کو مزید پریشانی نہ ہو۔	612

8	**تجاویز:**	
801	اس مرکزِ صحت میں آپ کے خیال میں مریض اور عملے کے درمیان تعلق کو بہتر بنانے کے لیے کس قسم کے اقدامات کرنے کی ضرورت ہے؟ کس کی طرف سے ایسے اقدامات کیے جانے چاہیے اور کیسے؟ اگر آپ سے پوچھا جائے تو آپ کیا کریں گے؟	**علیحدہ پوچھیں:**<br>- عملہ کی صلاحیتوں کو بڑھانے کے لیے<br>- کام کے اخلاقیات<br>- ٹیم کے درمیان بہتر ربط<br>- مرکز صحت کی پالیسی<br>- سپروائز کرنا<br>- آپ کو ایسا کیوں لگتا ہے؟<br>- یہ تبدیلیاں اس مرکز صحت میں کس طرح کی جا سکتی ہیں؟
802	آپ کے خیال میں عملے کو دیکھ بھال فراہم کرنے کے دوران کن چیزوں کا خیال رکھنا چاہیے جس سے حاملہ خواتین کو محسوس ہو کہ ان کا عزت و احترام کے ساتھ علاج کیا جا رہا ہے؟	
803	آپ کے خیال میں کون سا طریقہ سب سے بہترین ہے جس سے اس بات کی یقین دہانی کی جائے کو تمام خواتین کو عزت و احترام کے ساتھ دیکھ بھال فراہم کی جائے؟	

Supplement to: Asim M, Hameed W, Khan B, Saleem S, Avan BI. Applying the COM-B model to understand the drivers of mistreatment during childbirth: a qualitative enquiry among maternity care staff. *Glob Health Sci Pract*. 2022;10(6):e2200267. https://doi.org/10.9745/GHSP-D-22-00267

SUPPLEMENT 2. TOOLS OF PARTICIPATORY REFLECTIVE PRACTICES

Timeline diagram of daily routine work of service provider

Name of service provider ______________

	Morning shift				Evening shift				Night shift			
Days	8-09 AM	10-11AM	12-01PM	02-03PM	04-05 PM	06-07 PM	08-09 PM	10-11 PM	12-01AM	02-03AM	04-05AM	06-06 AM
Monday												
Tuesday												
Wednesday												
Thursday												
Friday												
Saturday												
Sunday												

Supplement to: Asim M, Hameed W, Khan B, Saleem S, Avan BI. Applying the COM-B model to understand the drivers of mistreatment during childbirth: a qualitative enquiry among maternity care staff. *Glob Health Sci Pract*. 2022;10(6):e2200267. https://doi.org/10.9745/GHSP-D-22-00267

Flow diagram to capture the opportunities and challenges faced by the service providers to provide respectful care

(Provider's Name)'s flow diagram

Flow diagram: A typical day

Stages of day				
Steps within day				
Distribution of work				
Challenges				
Opportunities				

Supplement to: Asim M, Hameed W, Khan B, Saleem S, Avan BI. Applying the COM-B model to understand the drivers of mistreatment during childbirth: a qualitative enquiry among maternity care staff. *Glob Health Sci Pract*. 2022;10(6):e2200267. https://doi.org/10.9745/GHSP-D-22-00267

SUPPLEMENT 3. SUMMARY OF BEHAVIOURAL DRIVERS OF PROVIDERS NOT PRACTICING RESPECTFUL MATERNITY CARE

**Themes**		**Nonclinical staff**	**Clinical staff**
Capability	Physical	• Lack of awareness about the psychosocial support of patients<br>• No training opportunities on RMC	• Lack of awareness about the psycho-social support of patients<br>• No training opportunities on RMC
	Psychological	• No realization of patient's rights at facility<br>• Lack of recognition of patient's differential needs	• Lack of understanding of patient's respect<br>• Lack of recognition of patient's differential needs
Opportunity	Physical	• Lack of nfrastructural support (e.g., curtains and separators for privacy in delivery room and labour ward)<br>• Shortage of drugs<br>• Job aids for RMC and guidelines were not present	• Lack of infrastructural support, (e.g., curtains and separators for privacy in delivery room and labor ward)<br>• Shortage of drugs<br>• No RMC guidelines available
	Social	• Lack of understanding the role of birth companions<br>• Attendant's roles are not defined<br>• Lack of team work	• No value clarifications for male birth companions<br>• Lack of team coordination<br>• Lack of policies for companion engagement<br>• Lack of attendant and patient compliance cause disrespect and abuse
Motivation	Reflective	• Personality traits of providers cause disrespect and abuse<br>• A mindset that patients are difficult, uncooperative, intolerant, and aggressive<br>• Normalized manifestations of mistreatment	• Personality traits of providers cause mistreatment and abuse<br>• Manifestations of mistreatment

Supplement to: Asim M, Hameed W, Khan B, Saleem S, Avan BI. Applying the COM-B model to understand the drivers of mistreatment during childbirth: a qualitative enquiry among maternity care staff. *Glob Health Sci Pract*. 2022;10(6):e2200267. https://doi.org/10.9745/GHSP-D-22-00267

	Automatic	• Feedback and complaint system is not functional<br>• Mechanism of accountability was not placed	• No functioning feedback and complaint system<br>• No mechanism of accountability<br>• No staff appraisal and incentivization system present
